# Anti-tumor necrosis factor-α therapy may not be safe during pregnancy in women with inflammatory bowel disease: an updated meta-analysis and systematic review

**DOI:** 10.1186/s12884-024-06443-w

**Published:** 2024-04-08

**Authors:** Wei Huang, Xinxing Zhang, Li Zhang, Xiaosong Dai, Heping Chen, Qin Xie

**Affiliations:** Department of Geriatric Medicine and Gastroenterology, Sichuan Provincial People‘s Hospital, School of Medicine, University of Electronic Science and Technology of China, Chengdu, China

**Keywords:** Inflammatory bowel disease, Tumor necrosis factor, Pregnancy, Biologic treatment, Adverse pregnancy outcomes

## Abstract

**Background:**

Inflammatory Bowel Disease (IBD) affects reproductive-aged women. Active disease can lead to decreased fertility. Although the vast majority of international guidelines recommend for the continuation of anti-TNF-α during pregnancy, recent studies have raised concerns about the safety of anti-tumor necrosis factor-α (TNF-α) therapy during pregnancy, both for patients and for physicians.

**Methods:**

Studies that evaluate the safety of anti-TNF-α therapy in pregnant women with IBD were identified using bibliographical searches. An updated meta-analysis was performed for pregnancy outcomes, such as live birth, abortion, still birth, preterm birth, low birth weight, congenital abnormalities, and neonatal infection. Odds ratio (OR) with 95% confidence interval (CI) are reported. Data on disease activity, timing of anti-TNF-α therapy were collected for further analysis.

**Results:**

Overall, 11 studies were screened from on-line databases and international meeting abstracts. An increased risk of abortion (OR, 1.33; 95% CI, 1.02–1.74; *P* = 0.04) and preterm birth (OR, 1.16; 95% CI, 1.05–1.28; *P* = 0.004), and a decreased risk of live birth (OR, 0.83; 95% CI, 0.74–0.94; *P* = 0.002]) were found in the anti-TNF-α therapy group compared with the control group (no use of anti-TNF-α therapy). The subgroup analyses based on the disease activity showed there is no significant association between the use of anti-TNF-α therapy during pregnancy on adverse pregnancy outcomes of abortion, preterm birth, and live birth. The rates of still birth, low birth weight, and congenital abnormalities in the anti-TNF-α therapy group were not significantly different from those in the control group.

**Conclusions:**

Anti-TNF-α therapy does not increase the risks of still birth, low birth weight, and congenital abnormalities; however it may be assicated with increased risks of abortion and preterm birth, which are accompanied by a lower rate of live birth. Although these findings may be confounding by potential disease activity, they offer some opposite viewpoints with biologic agent use. Therefore, more studies are required to further confirm the safety of anti-TNF-α therapy in pregnancy with IBD.

**Supplementary Information:**

The online version contains supplementary material available at 10.1186/s12884-024-06443-w.

## Background

Inflammatory bowel disease (IBD), comprising ulcerative colitis (UC) and Crohn’s disease (CD), is a chronic, relapsing inflammatory disorder of the intestinal tract that frequently affects people during their reproductive years [1–3]. Active disease causes an increased risk of abortion, preterm birth, low birth weight, and congenital abnormalities [4].Therefore, medical treatment is required to control active disease before and during pregnancy.

Anti-tumor necrosis factor-α (TNF-α) is a type of drug that blocks the action of TNF-α and neutralizes its biologic effect. Anti-TNF-α therapy is highly effective and is increasingly being used to induce remission and prevent flare-ups of IBD during pregnancy [5–7].The most commonly anti-TNF-α agents used in IBD are infliximab (IFX), adalimumab (ADA), certolizumab pegol(CZP), and golimumab. TNF-α is a cytokine associated with inflammation, and it is also important in regulating placental hormones and modulating trophoblast proliferation and invasion [8]. The dysregulation of TNF-α in pregnancy affects the pregnancy outcome, and it is related to complications, such as pregnancy loss and preterm labor [8, 9]. Although anti-TNF-α drugs were rated category B by the Food and Drug Administration and were recommended to be initiated or continued throughout pregnancy [10], this recommendation was mainly based on animal experimental data, and the clinical data are controversial. Previous meta-analyses did not show any increased risk of adverse outcomes in women with IBD during pregnancy who had anti-TNF-α therapy compared with the those not exposed to anti-TNF-α drugs [11, 12]. However, recent evidence has shown that the use of anti-TNF-α drugs is associated with adverse pregnancy outcomes (APOs) [13–15]. These APOs include birth defects without a distinct pattern of malformations, preterm birth, reduced birth weight, and maternal complications. Nevertheless, the findings of these studies may be influenced by various factors, such as the type of disease, disease activity, and the duration of anti-TNF-α drugs. Most of the available clinical data on anti-TNF-α drugs using in pregnancy are from observational studies because of the ethical constraints, and no adequate and well-controlled clinic trial of pregnant women has been conducted. Treatment options for pregnant women often lack strong evidence-based recommendations.

We performed an updated meta-analysis and systematic review to assess the safety of anti-TNF-α therapy during pregnancy in patients with IBD to better inform choices and clinical decision-making.

## Methods

### Date sources and search strategy

A systematic literature search was performed to identify studies that investigated the pregnancy outcomes in women with IBD on anti-TNF-α therapy. We searched electronic databases, such as MEDLINE, EMBASE, Web of Science, Cochrane Central Register of Controlled Trials from their inception to March 2023. The search strategy was performed with the following search terms as both MeSH terms and free-text terms: inflammatory bowel disease/IBD, ulcerative colitis/UC, Crohn’s disease/CD, pregnancy or pregnant, pregnancy outcomes, adalimumab/ADA, infliximab/IFX, certolizumab pegol/CZP, golimumab/GLM, or tumor necrosis factor (Supplementary Table e3). Additionally, meeting abstracts and the reference lists of retrieved articles were reviewed for additional relevant studies. No language restriction was imposed. This study was registered in PROSPERO (CRD42023405705) and reported following the PRISMA guidelines [16].

### Eligibility criteria

Randomized, controlled trials (RCTs), observational studies, and case series(*n* > 10) that evaluated pregnancy outcomes of women with IBD were eligible for the systematic review. Studies were further narrowed with women treated with anti-TNF-α drugs (i.e., IFX, ADA, certolizumab pegol, and golimumab) at any point during pregnancy. Pregnancy outcomes included the number of live births, still births, preterm birth (gestational age < 37 weeks), low birth weight (birth weight < 2500 g), abortion (spontaneous or elective), congenital abnormalities, and neonatal infection. Studies were excluded if there were insufficient data for extraction or a lack of appropriately matched controls (studies only include women with IBD exposed to anti-TNF-α therapy during pregnancy). If more than one study was published using the same study population, only the most recent study was included.

### Study selection

Two reviewers (Wei Huang and Xinxing Zhang) independently reviewed the title and abstract for study eligibility and performed the data extraction. The paper was reviewed if either one of the two investigators thought that an abstract was relevant. If there were any discrepancies regarding information provided in the title and abstract, the full article was reviewed for clarification. Differences in opinion were resolved by discussing with a third author (Qin Xie).

### Data extraction and quality assessment

All of the data were tabulated with standard data abstraction sheets. The following characteristics of each study and each type of intervention were extracted: authors, study design, publication year, intervention characteristics, numbers of pregnancies with IBD and controls, and pregnancy outcomes. In addition, information on disease activity, time of anti-TNF-α therapy, and neonatal infection were extracted.

A quality assessment was carried out using the Newcastle–Ottawa Scale [17], which is an assessment tool for observational studies that grades quality using three categories: selection of study groups, comparability of groups, and determination of exposure (case–control studies) or outcome (cohort studies). Studies scoring ≥ 7 on the 9-point scale were considered high quality.

### Statistical analysis

A meta-analysis was performed using the statistical tool Review Manager (version 5.4, Copenhagen, Denmark). Dichotomous data are expressed as odds ratio (OR) with 95% confidence interval (CI). A random or fixed effect model was selected according to the heterogeneity. I^2^ statistics were used to quantified the heterogeneity [18]. If I^2^ was > 50%, indicating significant heterogeneity among studies. Pooled OR were assessed using random-effects mode. A sensitivity analysis was performed to detect the effect of any one of the included studies on the overall estimate by excluding one of them according to the sample size. In the assessment of publication bias, a funnel plot was used if sufficient data were available [19]. It has been suggested that such analysis is only reliable when conducted on 10 or more studies.

## Results

### Search results

**Fig. 1:**
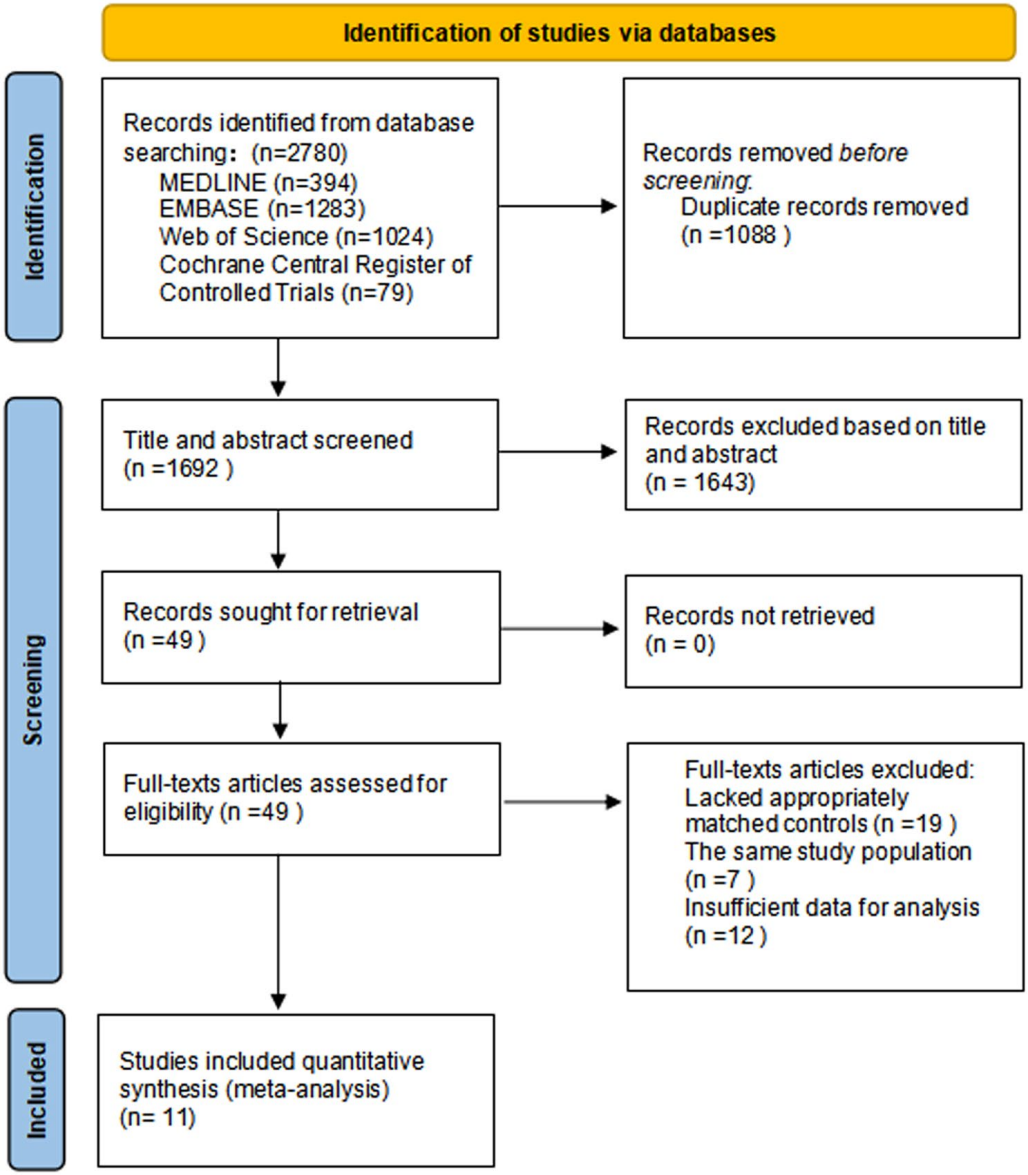
Study screening and selection flow diagram

We found 2780 articles using our search strategy. After removing duplicates, 1692 articles were screened for the title and abstract. Forty-nine articles met the eligibility and were further reviewed, of which 38 articles were excluded. Among them, 19 studies lacked appropriately matched controls, 7 studies used repeat population groups, and 12 studies lacked sufficient data for extraction. Finally,11 studies met the criteria for inclusion in the review [15, 20–29] (Fig. 1).

Overall, 41,028 pregnancies(which could involve multiple pregnancies from the same women) were included in the 11 studies (6625 pregnancies exposed to anti-TNF-α versus 34,403 unexposed pregnancies). Most studies reported data on abortion, preterm birth (PTB), and congenital anomalies (CA), except for those by Diav-Citrin et al [22] and Meyer et al [23], who provided data only for CA or PTB. Five studies mentioned low birth weight [20, 24–26, 28]. Six and four studies included data on live birth [15, 20, 21, 24, 27, 29] and still birth [20, 23–25], respectively. Four studies [15, 20, 25, 27] contained data on neonatal infection. The characteristics of the included studies and the quality of the evidence for the outcomes of the included studies are shown in Table 1.

**Table 1:** characteristics and quality of included studies in the meta-analysis

Author	Study design	Disease	Anti-TNFa agent	Control Group	Concomitant Medications		Timing of exposure	Age at conception (mean, year)		Duration of disease (mean, year)		Study quality
					Anti-TNFa group	Control Group		Anti-TNFa group	Control group	Anti-TNFa group	Control Group	
Johnson et al. [29]	p	CD	ADA	Not exposed to anti-TNFa therapy.	Not specified	Not specified	Not specified	Not specified	Not specified	Not specified	Not specified	*******
Schnitzler et al. [24]	R	CD, UC and indeterminate colitis	IFX, ADA	Not exposed to anti-TNFa during pregnancy or 3 months before.	5- ASA: 12/42 AZA/6MP : 22/42 Steroids: 3/42	Not specified	Within 3 months prior conception, and/or During pregnancy (up to end of 2nd trimester).	29	27	Not specified	Not specified	*******
Mahadevan et al. [28]	P	CD and UC	IFX, ADA, CZP	Not exposed to anti-TNFa therapy.	5-ASA, steroids, antibiotics	5-ASA, steroids, antibiotics	Not specified.	Not specified	Not specified	Not specified	Not specified	*******
Casanova et al. [25]	R	CD and UC	IFX, ADA, CZP	Not exposed to anti-TNFa during pregnancy or 3 months before.	AZA: 36/66, 6-MP: 1/66	5-ASA: 240/318, Steroids: 6/318, 5-ASA + Steroids:22/318	21% discontinued the treatment at conception, 20% during the first trimester, 29% during the second trimester, and 30% of pregnancies continued with anti-TNFα drugs during the third trimester of gestation.	Not specified	Not specified	Not specified	Not specified	*******
Seirafi et al. [15]	R + P	UC, CD and IBD unclassified	IFX, ADA, CZP	Not exposed to anti-TNFa during pregnancy or 3 months before.	AZA (16%), 6MP (1.5%), CsA (0.7%), 5ASA (4.5%), steroids (8%)	Thiopurines(39.5%), 5ASA and/or steroids (39.5%)	73% stopped preventively before week 30; 20% continued until delivery.	29.3	28.9	9.3	9.9	********
Diav-Citrin et al. [21]	P	CD and UC	IFX, ADA	Not exposed to anti-TNFa during pregnancy or 3 months before.	Not specified	Not specified	Not specified	Not specified	Not specified	Not specified	Not specified	********
Komoto et al. [26]	R	CD and UC	IFX, ADA	Pregnancies not exposed to anti-TNFa.	AZA(8/34) Steroids + AZA(2/34)	AZA (6/38), 5ASA (28/38), 6MP (1/38)	During pregnancy	31.2	30.1	6.6	7.6	*******
Lichtenstein et al. [20]	P	CD	IFX	Not exposed to anti-TNFa therapy during pregnancy	5-ASA, Steroids, Immunosuppressive drugs, Antibiotics	5-ASA, Steroids, Immunosuppressive drugs, Antibiotics	Exposure ≤ 365 days before the date of the delivery	Not specified	Not specified	6.1	6.6	*******
Luu et al. [19]	R	CD	IFX, ADA, CZP, golimumab	Pregnancies not exposed to anti-TNFα	AZA: 198/1457 MCT: 25/1457	AZA: 1256/9819 MCT: 50/9818	18.3% stopped before week 12, 33.8% stopped between week 12–24, 48% continued after 24 weeks, 31.6% continued after 37 weeks.	29.4	31	Not specified	Not specified	******
Moens et al. [27]	R	CD and UC	IFX, ADA	Pregnancies unexposed to immunomodulatory and biologic	Immunomodulators (39/186) Steroids (14/186)	Steroids (25/184)	For IFX: 20% discontinue during 1st trimester, 39% during 2nd trimester, 13% during 3rd trimester, 28% continued throughout pregnancy. For ADA: 8% continued during 1st trimester, 32% during 2nd trimester, 18% during 3rd trimester, 23% continued throughout pregnancy.	30	31	7	6	*******
Meyer et al. [23]	R	CD and UC	IFX, ADA, CZP, golimumab	Pregnancies not exposed to anti-TNFα	Thiopurines (839/4364) Steroids (1233/4364)	Thiopurines (3554/23,365) Steroids (5387/23,365)	14.5% during the trimester before pregnancy, 13.3% during 1st trimester, 11.3% during 2nd trimester, 3.4% during 3rd trimester	29	30	4.3	5.6	******

ADA adalimumab, CZP certolizumab pegol, CD Crohn’s disease, IFX infliximab, P prospective, R retrospective, TNFα tumor necrosis factorα, UC ulcerative colitis, 5-ASA 5-aminosalicylic acid, 6-MP 6-mercaptopurine, AZA azathiopurine, MCT mercaptopurine

### Abortion and live birth

**Fig. 2:**
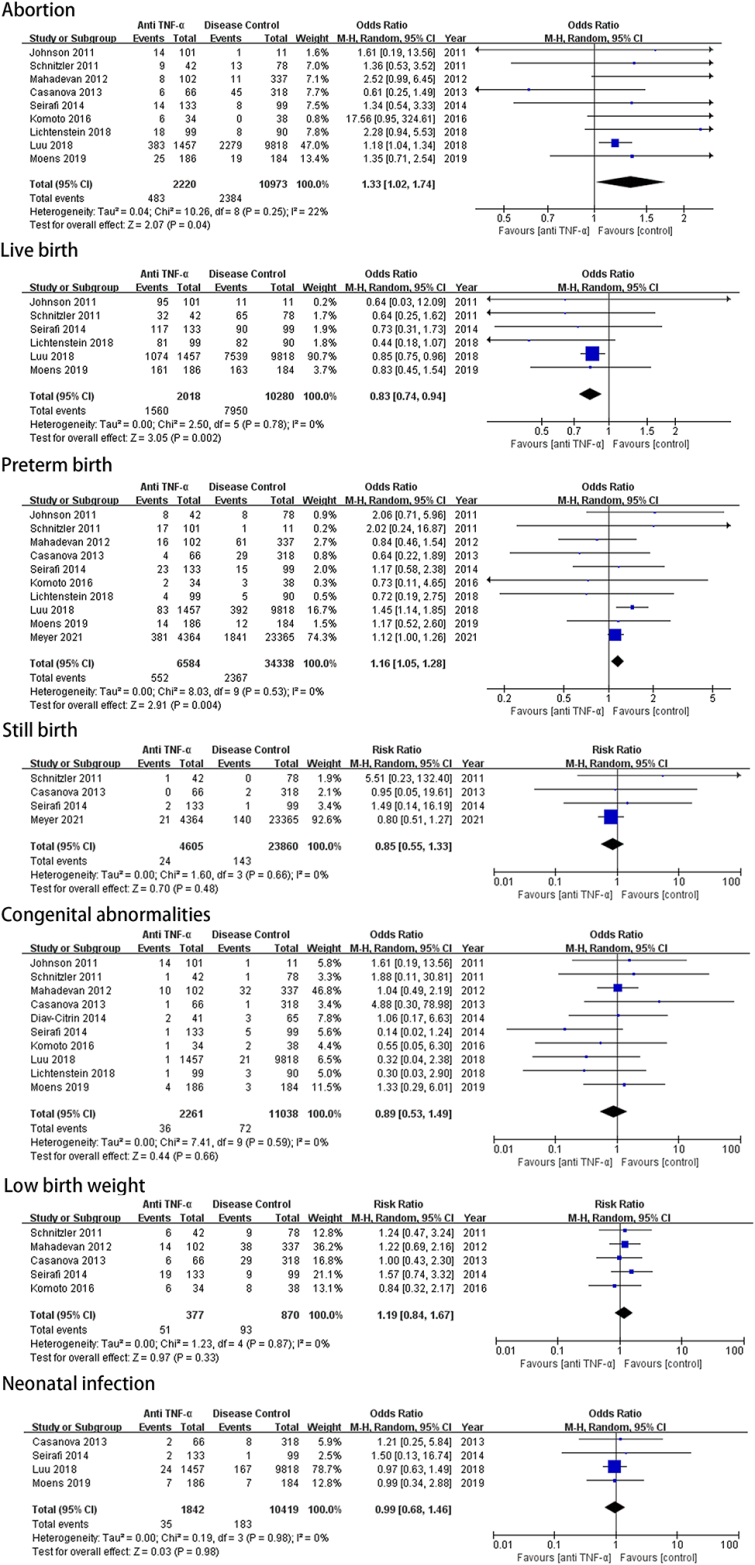
Forest plot of studies assessing pregnancy outcomes among patients on anti-TNFa compared with control groups

Nine and six studies met the criteria for inclusion for abortion [15, 20, 21, 24–29] and live birth, respectively. Individual studies for either outcome did not reach statistical significance, except for the study by Luu et al [15]. Patients with IBD on anti-TNF-α therapy showed an increased risk of abortion (OR, 1.33; 95% CI, 1.02–1.74; *P* = 0.04) and a decreased rate of live birth (OR, 0.83; 95% CI, 0.74–0.94; *P* = 0.002) compared with controls (Fig. 2). The participants in the study by Luu et al. had the heaviest weight; therefore, we investigated the effect of this study on the pooled results by excluding it. The difference became no longer significant, and the combined ORs were 1.49 (95% CI, 1.00–2.21; *P* = 0.05) and 0.68 (95% CI, 0.46–1.00; *P* = 0.05), respectively.

### Preterm birth and still birth

Data on the rates of preterm birth and still birth were abstracted and analyzed. Ten studies [15, 20, 21, 23–29] reported PTB outcomes of pregnancy in IBD, and these included 6584 events in women who received anti-TNF-α therapy and 34,338 events in disease-matched controls. The pooled OR was significantly increased (OR, 1.16; 95% CI, 1.05–1.28; *P* = 0.004). Four studies reported still birth outcomes in pregnant women with IBD, and the pooled OR was 0.85 (95% CI, 0.55–1.33; *P* = 0.48) when patients treated with anti-TNF-α therapy (*n* = 4605) were compared with disease-matched controls (*n* = 23,860) (Fig. 2).

### Congenital abnormalities and low birth weight

Ten studies [15, 20–22, 24–29] included data of CA. The pooled OR for CA was 0.89 (95% CI, 0.53–1.49; *P* = 0.66), with no significant difference between the treatment and control groups(Fig. 2). Similar results were found in a pooled analysis of the studies of low birth weight (OR, 1.19; 95% CI, 0.84–1.67; *P* = 0.33). Additionally, Schinitzler et al. [24] found that intensifying IFX treatment (10 mg/kg IFX, only in trimesters 1 and 2) in women with IBD experiencing a flare during pregnancy was not associated with the occurrence of malformations in children.

### Disease activity

**Fig. 3:**
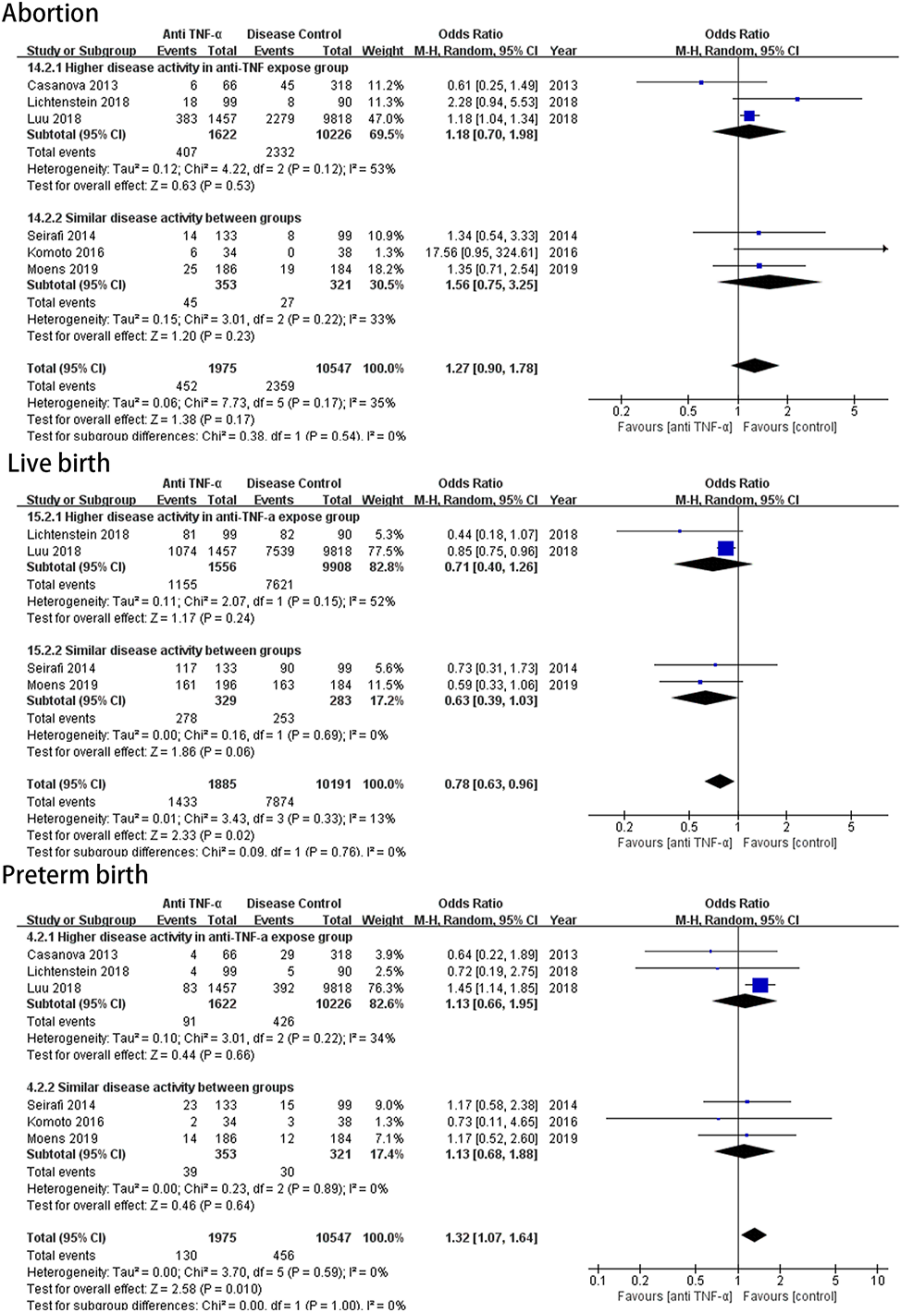
Forest plot for subgroups analysis of abortions, live birth and preterm birth stratificate by disease activity

Six studies included data on disease activity(Supplementary Table 2). The indices used for disease activity in these studies showed heterogeneity. Therefore, we were not able to perform a meta-analysis. Three of the six studies showed that anti-TNF-α exposed group exhibited higher disease activity during pregnancy and were categorized as the “Higher disease activity in anti-TNF expose group”. These studies were by Luu et al [15] (21.4% vs. 4.3% moderate-to-severe disease), Lichtenstein et al. [21] (10.6% vs. 2.67% at conception and 17.2% vs. 3.4% moderate-to-severe disease during pregnancy, and Casanova et al. [25] (27.3% vs. 9.7% at conception and 34.8% vs. 18.6% during pregnancy).The other three studies by Komoto et al. [26], Seirafi et al. [20], and Moens et al. [27] showed no statistically difference in disease activity between groups. These studies were categorized into the “Similar disease activity between groups”. Further subgroup analysis revealed that differences in abortion, PTB and live birth between the two groups were independent of whether the TNF-α group included more active patients (Fig. 3).

### Timing of anti-TNF-α therapy

As shown in Table 1, eight studies [15, 20, 21, 23–27] included data on the timing of anti-TNF-α therapy. Additionally, four of these studies [15, 20, 24, 25] analysis the effect of duration of exposure during pregnancy on pregnancy outcomes. Schinitzler et al. [24] reported anti-TNF-α agents use in trimesters 1 and 2, but not in trimester 3, and no increased risk of APOs was found. Seirafi et al. [20] reported that discontinued treatment of anti-TNF-α agents before week 30 resulted in 14% of flare during the last trimester of pregnancy with APOs and 32% of flare during the early post-partum period. While, in those continued therapy, the fate of flare during the early post-partum period was 26%. Similarly, Casanova et al. [25] demonstrated no difference in APOs between pregnancies in which anti-TNF-α drugs were suspended during the second trimester and pregnancies in which this therapy was performed during the third trimester (31.6% vs. 25%). However, pregnancies in which anti-TNF-α drugs were suspended during the first trimester showed a higher frequency of APOs than pregnancies that were exposed to anti-TNF-α drugs during the three trimesters of pregnancy (69% vs. 25%). Additionally, suspension of anti-TNF-α drugs during the first trimester resulted in a higher IBD activity rate (39% vs. 25%) and higher frequency of spontaneous abortion (46% vs. 0%). Maintaining anti-TNF-α after 24 weeks did not increase the risk of maternal complication, but interrupting the anti-TNF-α increased relapse risk [15].

### Risk of neonatal infection

Four studies included data on neonatal outcomes with a risk of infection. The neonatal infection rate ranged from 1.5 to 3.8% in the anti-TNF-α group, whereas it ranged from 1 to 3.8% in the non-exposed group. None of the studies show a significant difference in the risk of infection between the groups, and the pooled OR was 0.99 (95% CI, 0.68–1.46; *P* = 0.98) (Fig. 2).

### Publication bias and heterogeneity

The publication bias of pregnancy outcomes (live birth, still birth, abortion, preterm birth, low birth weight, congenital abnormalities) was also assessed using funnel plots. The funnel plot seems symmetric except for live birth and still birth (Supplementary Fig. 1). Because of less than ten studies were included, no conclusive conclusion regarding publication bias can be made except for CA and PTB.

Heterogeneity tests were performed for all pregnancy outcomes measured. There was no significant heterogeneity in any of the adverse outcomes (Fig. 2).

## Discussion

In contrast to previous meta-analyses, this meta-analysis suggested that anti-TNF-α therapy may be associated with a higher risk of abortion and PTB, accompanied by a lower rate of live birth. However, no evidence shows that anti-TNF-α therapy was associated with still birth, low birth weight, congenital abnormalities, or neonatal infection in the meta-analysis. This is the first meta-analysis to show that anti-TNF-α therapy may not be safe during pregnancy in women with IBD.

Due to concerns about the adverse effects of disease and therapeutic drugs, there has been an increase in voluntary childlessness among women with IBD [30]. A considerable number of patients with anti-TNF-α therapy also have APOs owing to disease recurrence after drug withdrawal during pregnancy [15]. The adverse effects and duration of anti-TNF-α drugs are a major concern for pregnant women and their physicians. In 2014, Narula et al. [11] conducted a meta-analysis to compare APOs (e.g. abortion, CAs, PTB, and low birth weight) between the anti-TNF-α therapy group and the disease-matched control group. They found no increased risk of APOs between the two groups. Shihab et al. [12] performed another meta-analysis in 2016 and showed the same results. The main reason for the differences between our meta-analysis and previous meta-analyses may be that we included more recent studies on this topic, which resulted in a larger pooled population size. The addition of more studies in meta-analyses decreases between-study variations and increases power [31]. Our results are partly consistent with a recent meta-analysis that showed a significant risk of preterm birth in women with immune-mediated inflammatory disease (including IBD) who were exposed to anti-TNF-α agents during pregnancy [32]. A population-based study from Denmark, Finland, and Sweden also showed that anti-TNF-α agents were associated with increased risks of preterm birth, cesarean section, and small for gestational age [33]. Nevertheless, after adjusting for markers of disease severity, the association between anti-TNF-α and preterm birth was attenuated, suggesting residual confounding by disease severity.

It’s known that disease activity is associcated with APOs [4]. To analysis the effect of disease activity on the differences found in abortion, PTB and live birth in our study, we performed a subgroup analysis based on the disease activity between the exposed and control groups. The results showed there was no association between the use of anti-TNF-α therapy during pregnancy and APOs such as abortion, PTB, and live birth, regardless of whether disease activity was higher in the anti-TNF-α group. It is consistent with a recent cohort study, which showed that after adjusting for disease activity, there was no increase in adverse pregnancy and neonatal risks in anti-TNF-α exposed pregnancies [34]. However, due to the limited number of studies available for subgroup analysis, the results of the subgroup analysis may not accurately reflect the actual risk of anti-TNF-α therapy.

Another concerning is whether or not to continue with anti-TNF-α in the third trimester. Except for certolizumab, other anti-TNF-α agents readily traverse the placenta via active transport [35]. There is a theoretical risk of increased infections in the neonate owing to a reduced immune status in newborns of women exposed to anti-TNF-α during pregnancy [36]. Besides, there was study showed continuation of anti-TNF-α therapy after gestational week 30 were independently associated with lower birth weight [37]. Therefore, the Toronto and European consensus recommends administering the last dose at 22–24 weeks gestation for those at low-risk for an IBD relapse [38, 39]. However, despite the high percentage of the study population being exposed to anti-TNF-α during the third trimester in our study, the pooled data showed no increased risk of either neonatal infection or or low birth weight. This finding is in contrast to a previous meta-analysis, which showed an increased risk of low birth weight and infections in newborns of women with immune-mediated inflammatory disease exposed to anti-TNF-α agents [32]. Whether the disease type, medication duration and dose, or difference in concomitant medication is responsible for these differences needs to be determined by more clinical studies in the future.

Similar to all meta-analyses, this meta-analysis had several limitations. First, all studies were observational and some of the studies were retrospective in design. The statistical combination of data might have been subject to potential confounding factors, such as concomitant medication, disease activity, and paternal diseases. Although the majority of the included studies provided data on concomitant medications, the type and proportion of concomitant medications varied across studies, rendering the data unsuitable for subgroup analysis. The concomitant medications used for treating IBD in both exposed and control groups of the included studies were 5-aminosalicylic acid, Steroids and Immunomodulators, all of which were deemed to pose low risk during pregnancy [40]. Secondly, the studies included in our meta-analysis did not provide sufficient data on the type and dose of anti-TNF-α agents to analyze the relationship between specific agents or doses and APOs. In addition, we could not analyze the total unfavorable pregnancy outcomes in the studies because the definition and components were not consistent among the studies. Despite the above limitations, the strength of our study is the large pooled population size and the relatively low heterogeneity across studies. Besides, this meta-analysis does provide a comparison of risk against the composite of other IBD drugs in current use.

In conclusion, this meta-analysis suggests that anti-TNF-α therapy during pregnancy may be associated with increased rate of abortion and PTB. The rate of live birth in women who have anti-TNF-α therapy is lower than that in those who are not exposed to anti-TNF-α agents. Although these findings may be confounding by potential disease activity, they offer some opposite viewpoints with biologic agent use. More specific studies on the medication duration, dose, and specific agents in relation to pregnancy outcomes are required. These studies will help determine the sources of risk and help clinicians balance the risks of disease activity and drug side effects to select more appropriate treatment for pregnant women with IBD.

### Electronic supplementary material

Below is the link to the electronic supplementary material.

Supplementary Material 1

Supplementary Material 2

Supplementary Material 3

Supplementary Material 4

Supplementary Material 5

## Data Availability

The original contributions presented in the study are included in the article/supplementary material, further inquiries can be directed to the corresponding author/s.
