# Supplementary material for: Anti-tumor necrosis factor-α therapy may not be safe during pregnancy in women with inflammatory bowel disease: an updated meta-analysis and systematic review
Source: BMC Pregnancy Childbirth. 2024 Apr 8;24:251. doi: 10.1186/s12884-024-06443-w (PMC11000337; doi:10.1186/s12884-024-06443-w)
Supplement: Supplementary file 1 — Supplementary Material 1 [file 12884_2024_6443_MOESM1_ESM.docx]

Supplementary figure 1. Funnel plot for detecting publication bias of studies that report pregnancy-related outcomes

| 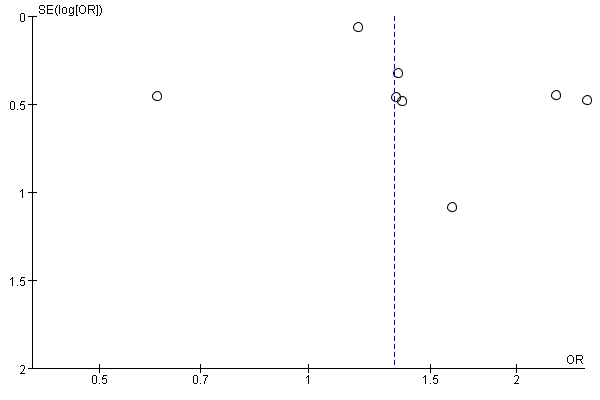Abortion | 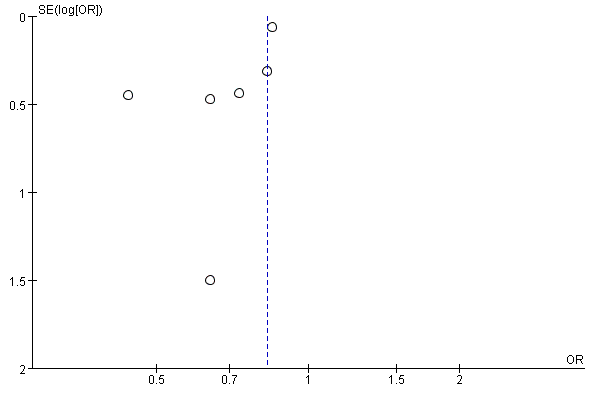Live birth |
| --- | --- |
| 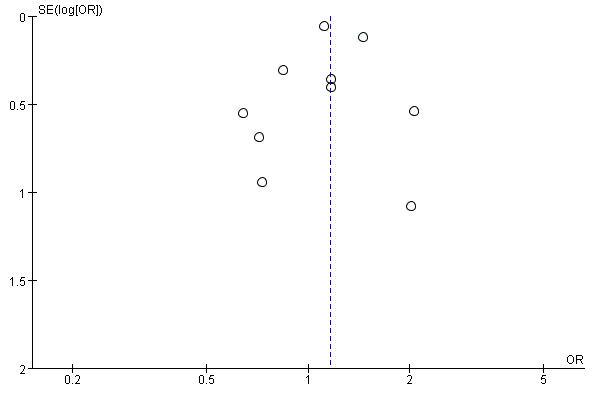Preterm birth | 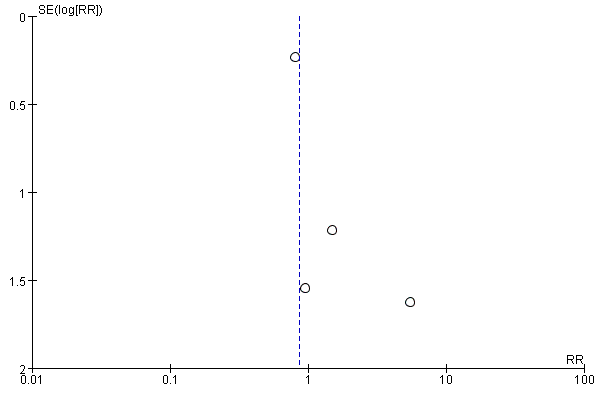Still birth |
| 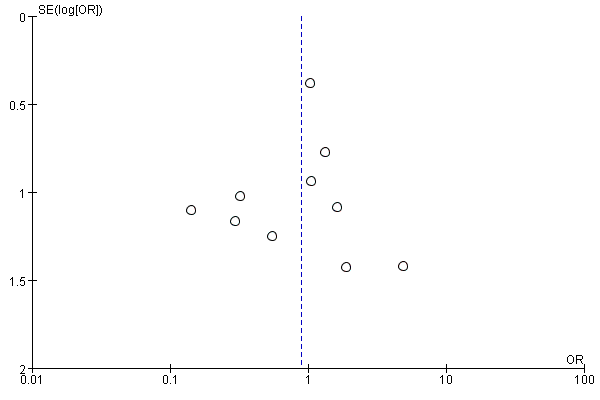congenital abnormalities | 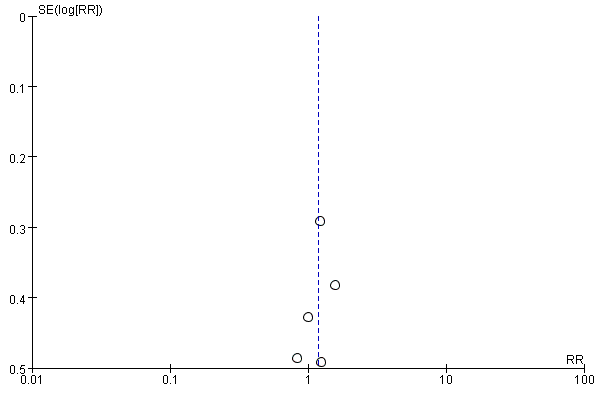Low birth weight |
| 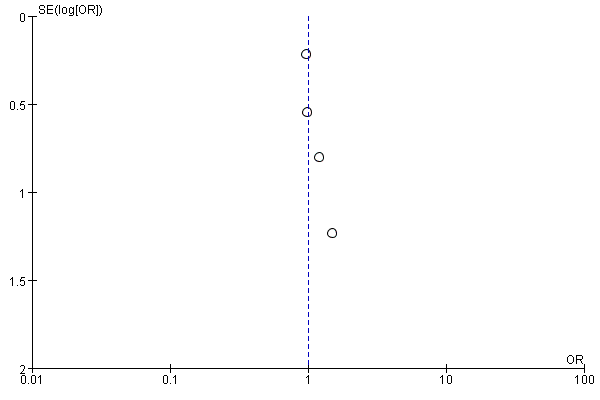Neonatal infectio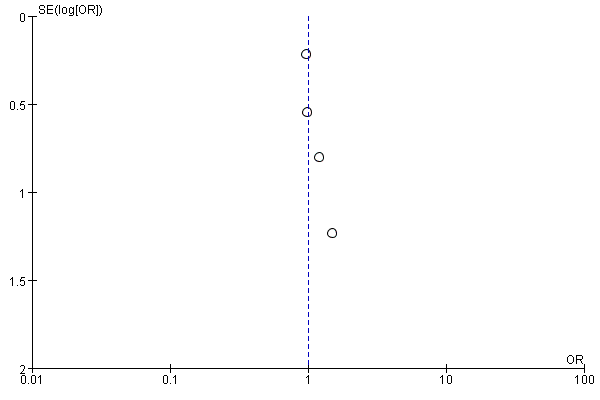ns |  |
