# Supplementary material for: Anti-tumor necrosis factor-α therapy may not be safe during pregnancy in women with inflammatory bowel disease: an updated meta-analysis and systematic review
Source: BMC Pregnancy Childbirth. 2024 Apr 8;24:251. doi: 10.1186/s12884-024-06443-w (PMC11000337; doi:10.1186/s12884-024-06443-w)
Supplement: Supplementary file 2 — Supplementary Material 2 [file 12884_2024_6443_MOESM2_ESM.docx]

Supplementary table 1. Definitions of abortion, preterm birth, stillbirth, and low birthweight for each study

| Author | definitions of   abortion | definitions of preterm birth | definitions of  stillbirth | definitions of   low birth weight |
| --- | --- | --- | --- | --- |
| Johnson  et al.29 | spontaneous or elective abortion | gestational age <37 weeks | / | / |
| Schnitzler  et al.24 | spontaneous or elective abortion | gestational age <37 weeks | Not specified | birth weight<2500g |
| Mahadevan  et al.28 | Spontaneous abortion prior to 20 weeks’ gestation. | gestational age <37 weeks | / | birth weight<2500g |
| Casanova  et al.25 | Spontaneous or elective abortions | gestational age of < 37 weeks at the moment of delivery | Not specified | neonatal weight under 2500g |
| Seirafi  et al.15 | Foetal loss prior to 20 weeks post-conception excluded elective abortion | delivery before 37 GW (gestational weeks) | Foetal loss after 20 weeks post-conception. | birth weight less than 2500 g |
| Komoto  et al.26 | Spontaneous abortion | birth at less than 35 weeks gestational age | / | Not specified |
| Lichtenstein  et al.20 | Spontaneous or elective abortions | Not specified | / | Not specified |
| Luu  et al.19 | Pregnancy loss | before the 37th week of amenorrhea | / | / |
| Moens  et al.27 | Early miscarriages  and elective terminations | delivery before 37 weeks of gestation | Foetal loss after 20 weeks post-conception | A birthweight less than 2500 g in a child born at term |
| Meyer  Et al.23 | / | before the 37week of amenorrhea | delivery of a dead fetus after 22week of amenorrhea | / |
