# Supplementary material for: Anti-tumor necrosis factor-α therapy may not be safe during pregnancy in women with inflammatory bowel disease: an updated meta-analysis and systematic review
Source: BMC Pregnancy Childbirth. 2024 Apr 8;24:251. doi: 10.1186/s12884-024-06443-w (PMC11000337; doi:10.1186/s12884-024-06443-w)
Supplement: Supplementary file 3 — Supplementary Material 3 [file 12884_2024_6443_MOESM3_ESM.docx]

Supplementary table 2. Disease activity in each study and indices used for assessment.

| Author | Disease activity | | Disease activity indices |
| --- | --- | --- | --- |
|  | Anti-TNFa group | Control Group |  |
| Casanova  et al.25 | At conception: 27.3%  During pregnancy: 34.8% | At conception: 9.7%*  During pregnancy: 18.6%* | For CD: the Harvey – Bradshaw index;  For UC: the Partial Mayo Score. |
| Seirafi  et al.15 | Active disease (HB ≥4 or Montreal ≥S1): 31% | Active disease (HB ≥4 or Montreal ≥S1): 17% | For CD: the Harvey – Bradshaw index;  For UC and and IBD Unclassified: the Montreal score. |
| Komoto  et al.26 | For CD: Bio only(1.9), Bio and IM(4.2);  For UC: Bio and IM(2.2) | For CD: Bio- IM-(2.6) ,IM only(3.6),  For UC: Bio- IM-(0.9), IM only(0.7) | For CD: the Harvey – Bradshaw index;  For UC: the Partial Mayo Score |
| Lichtenstein  et al.20 | At conception: 10.6%  During pregnancy: 17.2% | At conception: 2.6%*  During pregnancy: 3.4%* | Not specified |
| Luu  et al.19 | Mild: 78.6%  Moderate: 11.9%  Severe: 9.5% | Mild: 95.7%*  Moderate: 3%*  Severe: 1.3%* | A quantitative risk score, ranging from 0 to 13, and stratifying the risk as mild (0–1), moderate (2–4), and severe (5–13). |
| Moens  et al.27 | At conception: 17%  First trimester: 19%  Second trimester: 15%  Third trimester: 13% | At conception: 24%  First trimester: 22%  Second trimester: 25%  Third trimester: 25% | based on physician global assessment (PGA) as well as patients' recorded disease activity |

*: p<0.05 vs. Anti-TNFa group;

Bio- IM-: Pregnancies received neither anti-TNF or thiopurine therapy;

Bio only: Pregnancies received anti-TNF therapy only;

IM only: Pregnancies received thiopurine therapy only;

Bio and IM: Pregnancies received both anti-TNF and thiopurine therapy.
