# Supplementary material for: Anti-tumor necrosis factor-α therapy may not be safe during pregnancy in women with inflammatory bowel disease: an updated meta-analysis and systematic review
Source: BMC Pregnancy Childbirth. 2024 Apr 8;24:251. doi: 10.1186/s12884-024-06443-w (PMC11000337; doi:10.1186/s12884-024-06443-w)
Supplement: Supplementary file 4 — Supplementary Material 4 [file 12884_2024_6443_MOESM4_ESM.docx]

Supplementary table 3. The search strategy for each database.

| **MEDLINE** | #1 "Inflammatory Bowel Diseases"[MeSH Terms]  #2 "crohn*"[Title/Abstract] OR "IBD"[Title/Abstract] OR "inflammatory bowel disease*"[Title/Abstract] OR "regional ileitis"[Title/Abstract] OR "Colitis"[Title/Abstract]  #3 #1 OR #2  #4 "Pregnancy"[MeSH Terms]  #5 "pregnan*"[Title/Abstract]  #6 #4 OR #5  #7 "Tumor Necrosis Factor Inhibitors"[MeSH Terms] OR "tumor necrosis factor*"[Title/Abstract] OR "tnf"[Title/Abstract]  #8 "infliximab"[MeSH Terms] OR "infliximab"[Title/Abstract] OR "monoclonal antibody ca2"[Title/Abstract] OR "remicade"[Title/Abstract] OR "IFX"[Title/Abstract]  #9 "Adalimumab"[MeSH Terms] OR "Adalimumab"[Title/Abstract] OR "Humira"[Title/Abstract] OR "Exemptia"[Title/Abstract] OR "ADA"[Title/Abstract]  #10 "certolizumab pegol"[MeSH Terms] OR "Cimzia"[Title/Abstract] OR "CDP870"[Title/Abstract] OR "cdp 870"[Title/Abstract] OR "CZP"[Title/Abstract]  #11 "golimumab"[Title/Abstract] OR "CNTO-148"[Title/Abstract] OR "CNTO-148"[Title/Abstract] OR "Simponi"[Title/Abstract]  #12 or/7‐11  #13 #3 AND #6 AND #12 |
| --- | --- |
| **EMBASE** | #1. 'inflammatory bowel disease'/exp  #2. (crohn*:ab,ti OR ibd:ab,ti OR 'inflammatory bowel disease*':ab,ti OR 'regional ileitis':ab,ti) AND  colitis:ab,ti  #3. #1 OR #2  #4. 'pregnancy'/exp  #5. pregnan*:ab,ti  #6. #4 OR #5  #7. 'tumor necrosis factor inhibitor'/exp  #8. 'tumor necrosis factor*':ab,ti OR tnf*:ab,ti  #9. #7 OR #8  #10. 'infliximab'/exp  #11. infliximab:ab,ti OR 'monoclonal antibody ca2':ab,ti OR remicade:ab,ti OR ifx:ab,ti  #12. #10 OR #11  #13. 'adalimumab'/exp  #14. adalimumab:ab,ti OR humira:ab,ti OR exemptia:ab,ti OR ada:ab,ti  #15. #13 OR #14  #16. 'certolizumab pegol'/exp  #17. cimzia:ab,ti OR cdp870:ab,ti OR 'cdp 870':ab,ti OR czp:ab,ti  #18. #16 OR #17  #19. 'golimumab'/exp  #20. 'cnto 148':ab,ti OR simponi:ab,ti  #21. #19 OR #20  #22. #9 OR #12 OR #15 OR #18 OR #21  #23. #3 AND #6 AND #22 |
| **Web of Science** | (TS=Inflammatory bowel disease*  OR TS= crohn* OR TS=IBD OR TS=Regional ileitis OR TS=Colitis)  AND(TS=Pregnan*)AND(TS=Tumor Necrosis Factor* OR TS=TNF* OR TS= infliximab OR TS=etanercept OR TS=monoclonal antibody cA2 OR TS=remicade OR TS= IFX OR TS= Adalimumab OR TS=Humira OR TS=Exemptia OR TS=ADA OR TS=certolizumab pegol  OR TS= Cimzia OR TS=CDP870 OR TS=CDP 870 OR TS=CZP OR TS=golimuma  OR TS= CNTO-148 OR TS=CNTO 148  OR TS=Simponi) |
| **Cochrane Central Register of Controlled Trials** | #1 MeSH descriptor: [Inflammatory Bowel Diseases] explode all trees  #2 (Crohn*):ti,ab,kw OR (IBD):ti,ab,kw OR (Inflammatory bowel disease*):ti,ab,kw OR (Regional ileitis):ti,ab,kw OR (Colitis):ti,ab,kw  #3 #1 OR #2  #4 MeSH descriptor: [Pregnancy] explode all trees  #5 (Pregnan*):ti,ab,kw  #6 #4 OR #5  #7 MeSH descriptor: [Tumor Necrosis Factor Inhibitors] explode all trees  #8 (Tumor Necrosis Factor*):ti,ab,kw OR (TNF*):ti,ab,kw  #9 MeSH descriptor: [Infliximab] explode all trees  #10 (infliximab):ti,ab,kw OR (monoclonal antibody cA2):ti,ab,kw OR (remicade):ti,ab,kw OR (IFX):ti,ab,kw  #11 MeSH descriptor: [Adalimumab] explode all trees  #12 (Adalimumab):ti,ab,kw OR (Humira):ti,ab,kw OR (Exemptia):ti,ab,kw OR (ADA):ti,ab,kw  #13 MeSH descriptor: [Certolizumab Pegol] explode all trees  #14 (Cimzia):ti,ab,kw OR (CDP870):ti,ab,kw OR (CDP 870):ti,ab,kw OR (CZP):ti,ab,kw  #15 (golimumab):ti,ab,kw OR (CNTO-148):ti,ab,kw OR (CNTO 148):ti,ab,kw OR (Simponi):ti,ab,kw  #16 #7 OR #8 OR #9 OR #10 OR #11 OR #12 OR #13 OR #14 OR #15  #17 #3 AND #6 AND #16 |
