# Supplementary material for: Anti-tumor necrosis factor-α therapy may not be safe during pregnancy in women with inflammatory bowel disease: an updated meta-analysis and systematic review
Source: BMC Pregnancy Childbirth. 2024 Apr 8;24:251. doi: 10.1186/s12884-024-06443-w (PMC11000337; doi:10.1186/s12884-024-06443-w)
Supplement: Supplementary file 5 — Supplementary Material 5 [file 12884_2024_6443_MOESM5_ESM.docx]

Supplementary table 4. The quality assessment for the included studies.

| Author | selection | | | | Comparability | Outcome | | | Study quality |
| --- | --- | --- | --- | --- | --- | --- | --- | --- | --- |
|  | Representative of the exposed cohort | Selection of the non-exposed cohort | Ascertainment of exposure | Demonstration that outcome of interest was not present at start of study | Comparability of cohorts on the basis of the design or analysis | Assessment of outcome | Was follow-up long enough for outcomes to occur | Adequacy of follow-up of cohorts |  |
| Johnson  et al.29 | 1 | 1 | 1 | 1 | 0 | 1 | 1 | 1 | ******* |
| Schnitzler  et al.24 | 1 | 1 | 1 | 1 | 0 | 1 | 1 | 1 | ******* |
| Mahadevan  et al.28 | 1 | 1 | 1 | 1 | 1 | 1 | 0 | 1 | ******* |
| Casanova  et al.25 | 1 | 1 | 1 | 1 | 0 | 1 | 1 | 1 | ******* |
| Seirafi  et al.15 | 1 | 1 | 1 | 1 | 1 | 1 | 1 | 1 | ******** |
| Diav-Citrin et al.21 | 1 | 1 | 1 | 1 | 1 | 1 | 1 | 1 | ******** |
| Komoto  et al.26 | 1 | 1 | 1 | 1 | 1 | 1 | 0 | 1 | ******* |
| Lichtenstein  et al.20 | 1 | 1 | 1 | 1 | 0 | 1 | 1 | 1 | ******* |
| Luu  et al.19 | 1 | 1 | 1 | 1 | 0 | 1 | 1 | 0 | ****** |
| Moens  et al.27 | 1 | 1 | 1 | 1 | 1 | 1 | 1 | 0 | ******* |
| Meyer  Et al.23 | 1 | 1 | 1 | 1 | 0 | 1 | 1 | 0 | ****** |
